# Tauroursodeoxycholic acid alleviates secondary injury in spinal cord injury mice by reducing oxidative stress, apoptosis, and inflammatory response

**DOI:** 10.1186/s12974-021-02248-2

**Published:** 2021-09-20

**Authors:** Yonghui Hou, Jiyao Luan, Taida Huang, Tiancheng Deng, Xing Li, Zhifeng Xiao, Jiheng Zhan, Dan Luo, Yu Hou, Liangliang Xu, Dingkun Lin

**Affiliations:** 1 Department of Orthopedic Surgery, Guangdong Provincial Hospital of Chinese Medicine, The Second Affiliated Hospital of Guangzhou University of Chinese Medicine, No. 111 Dade Road, Guangzhou, 510120 Guangdong People’s Republic of China; 2 Guangzhou University of Chinese Medicine, No. 12, Jichang Road, Baiyun District, Guangzhou, 510405 Guangdong People’s Republic of China; 3 Lingnan Medical Research Center of Guangzhou University of Chinese Medicine, Guangzhou, 510405 Guangdong People’s Republic of China; 4 Research Center, The Seventh Affiliated Hospital of Sun Yat-sen University, Shenzhen, 518107 Guangdong People’s Republic of China; 5 Key Laboratory of Orthopaedics & Traumatology, The First Affiliated Hospital of Guangzhou University of Chinese Medicine, Guangzhou University of Chinese Medicine, Guangzhou, People’s Republic of China

**Keywords:** Tauroursodeoxycholic acid, Spinal cord injury, Oxidative stress, Inflammation

## Abstract

**Background:**

Tauroursodeoxycholic acid (TUDCA) is a hydrophilic bile acid derivative, which has been demonstrated to have neuroprotective effects in different neurological disease models. However, the effect and underlying mechanism of TUDCA on spinal cord injury (SCI) have not been fully elucidated. This study aims to investigate the protective effects of TUDCA in the SCI mouse model and the related mechanism involved.

**Methods:**

The primary cortical neurons were isolated from E16.5 C57BL/6 mouse embryos. To evaluate the effect of TUDCA on axon degeneration induced by oxidative stress in vitro, the cortical neurons were treated with H_2_O_2_ with or without TUDCA added and immunostained with Tuj1. Mice were randomly divided into sham, SCI, and SCI+TUDCA groups. SCI model was induced using a pneumatic impact device at T9-T10 level of the vertebra. TUDCA (200 mg/kg) or an equal volume of saline was intragastrically administrated daily post-injury for 14 days.

**Results:**

We found that TUDCA attenuated axon degeneration induced by H_2_O_2_ treatment and protected primary cortical neurons from oxidative stress in vitro. In vivo, TUDCA treatment significantly reduced tissue injury, oxidative stress, inflammatory response, and apoptosis and promoted axon regeneration and remyelination in the lesion site of the spinal cord of SCI mice. The functional recovery test revealed that TUDCA treatment significantly ameliorated the recovery of limb function.

**Conclusions:**

TUDCA treatment can alleviate secondary injury and promote functional recovery by reducing oxidative stress, inflammatory response, and apoptosis induced by primary injury, and promote axon regeneration and remyelination, which could be used as a potential therapy for human SCI recovery.

**Supplementary Information:**

The online version contains supplementary material available at 10.1186/s12974-021-02248-2.

## Introduction

Spinal cord injury (SCI) refers to complete or incomplete spinal motor and sensory dysfunction caused by injury to the spinal cord. SCI is a crippling disease, with high possibility resulting in disability of the patients, which brings a heavy burden to patients’ families and society. The pathophysiological process of SCI includes primary and secondary injuries [1–3]. The primary injury is caused by initial mechanical damage to the spinal cord which is an irreversible process [4, 5]. Subsequently, the ischemia and edema caused by hemorrhage, intervascular thrombosis, and vascular spasm will further exacerbate to the primary damage, which is usually called as secondary injury. The underlying basis of the secondary injury includes oxidative stress, inflammatory response, excitatory toxicity, cell apoptosis, and microcirculation disturbance [6, 7]. Therefore, inhibiting the progression of the second injury timely through attenuating oxidative stress and inflammation will benefit to reduce neuronal cell death and promote axon regeneration after primary injury, which could be an effective strategy to alleviate the neurological impairment of SCI [8, 9].

Tauroursodeoxycholic acid (TUDCA) is a hydrophilic bile acid which is the amino acid taurine conjugated with ursodeoxycholic acid [10, 11]. TUDCA has been approved by the US Food and Drug Administration (FDA) for the treatment of liver diseases such as cholestasis [12], cirrhosis, and hepatitis [13]. Besides, TUDCA can penetrate through the blood-brain barrier without any neuro- and cyto-toxicity and also has been reported to show protective effects against apoptosis, inflammation, oxidative stress, and/or mitochondrial dysfunction in different neurological diseases models, such as Parkinson’s disease (PD) [14–16], Huntington’s disease (HD), and Alzheimer’s disease (AD) [17, 18]. Thus, it attracts more research interests on its potential therapeutic effects on non-liver diseases, particularly on the neurological disease models [17, 19].

Previous studies have shown that TUDCA, as a chemical chaperone, had neuroprotective activities from different aspects, including anti-apoptosis, anti-inflammatory response, and anti-oxidative stress in different animal models of neurological diseases. For instance, Keene et al. reported that TUDCA treatment improved locomotor and sensorimotor recovery through reducing striatal atrophy, apoptosis, and the size of ubiquitinated neuronal intranuclear huntingtin inclusions in a transgenic HD animal model [15]. While in a PD mouse model induced by 1-methyl-4-phenyl-1,2,3,6-tetrahydropyridine (MPTP), TUDCA has been shown to ameliorate the motor symptoms by preventing the decrease of dopaminergic fibers and adenosine triphosphate (ATP) levels, as well as inhibiting mitochondrial dysfunction and neuroinflammation. In addition, Wu et al*.* reported that TUDCA treatment improved cognitive impairment and neurotoxicity induced by lipopolysaccharide (LPS) in mice through reducing the LPS-induced apoptosis and synaptic plasticity impairments [20]. In addition, TUDCA has also been shown to activate nuclear factor erythroid 2-related factor 2 (Nrf2) signaling, which can reduce the reactive oxygen species (ROS)-mediated damage, as well as enhance mitochondrial biogenesis and early neurogenesis [21].

Although there are strong evidences showing the protective properties of TUDCA in neurological diseases, few studies have reported the effects of TUDCA on the treatment of SCI and the underlying mechanism. Thus, we are aiming to investigate the effects of TUDCA on oxidative stress, inflammation, and apoptosis to evaluate the protective effects of TUDCA on axon regeneration and functional recovery in SCI treatment, and explore the underlying mechanism in this study.

## Materials and methods

### Animals

C57BL/6 mice were purchased from the Guangdong Medical Experimental Animal Center. The mice were placed in temperature-controlled conditions and supplied with standard rodent chow and water. All animal experiments were approved by the Ethics Committee of Guangzhou University of Chinese Medicine and performed according to the guidelines of the Chinese National Institutes of Health (Guangzhou, China, Certificate No. 44005800012426).

### TUDCA preparation

TUDCA used in this study (purity >98% of the total weight) was purchased from Shanghai Yuanye Bio-Technology Company Limited (Shanghai, China). TUDCA was dissolved in 0.9% normal saline.

### Primary culture of cortical neurons

Cortical neurons were extracted from embryos of pregnant C57BL/6 mice at embryonic day 16.5 (E16.5) according to the published protocol [22]. Shortly, the cerebral cortex was separated and cut into approximately 1-mm pieces in precooled Dulbecco’s modified Eagle medium: F-12 (DMEM/F12 medium, Gibco). Subsequently, the tissues were digested with 200 ug/ml papain (sigma, Beijing, China) for 25min at 37°C. After digestion with papain, the solution was filtered using a 100-μm cell strainer (BD Falcon) and centrifuged at 800rpm for 5min. The cell pellet was resuspended in complete Dulbecco’s modified Eagle medium (DMEM; Gibco) containing 10% fetal bovine serum (FBS, Gibco). Then, the cells were seeded into poly-D-lysine (PDL, sigma, Beijing, China) pre-coated 6-well plates, 24-well plates, or 96-well plates and incubated in 5% CO_2_ at 37°C. After 4 h, the cells were washed and maintained in Neurobasal medium (Gibco) containing 0.5mM L-glutamine (Gibco) and 2% B27 (B-27™ Supplement, Gibco). Half of the medium of the cortical neuron primary culture was changed every 3 days until the seventh day (7DIV). All cells used in the assay were cultured for three days. Cells were characterized under phase contrast microscope and with immunostaining of neuron-specific class III beta-tubulin (Tuj1) and neuronal nuclei (NeuN). The purity of cortical neurons in this primary culture was evaluated more than 90%.

### Cell viability assay

Cell viability was assessed using the Cell Counting Kit-8 (CCK-8, KeyGEN, China) assay according to the manufacturer’s protocol. Briefly, cortical neurons treated with TUDCA at the indicated concentrations were cultured for 48 h and simultaneously treated with H_2_O_2_ combined with or without TUDCA for 24 h. Before harvesting, CCK-8 solution was added into the culture medium and incubated at 37°C for 2 h. Finally, the absorbance of the culture medium at 450 nm was measured on a microplate reader (Bio-Rad) to determine the cell viability.

### ROS generation

Intracellular ROS generation was measured using DCFH-DA fluorescent probe (KeyGEN, China). With according treatments, cortical neurons were washed with PBS, and then 20 μM DCFH-DA fluorescent probe were added into the cultures with serum-free medium. Cells were further cultured at 37°C for 1 h according to the manufacturer’s instruction. ROS production in cells was determined by DCF fluorescence observed under fluorescence microscopy (Olympus IX73).

### LDH, total SOD, and GSH measurement

The biomedical kits (Jiancheng Institute of Biology, Nanjing, China) were used to measure and normalize the level of LDH, total SOD, and reduced GSH according to its manufacturer’s protocol.

### SCI model and treatment

The model of SCI was induced under sterile conditions and performed according to Allen’s method as previously described [23]. Before surgery, the mice were anesthetized using 1% pentobarbital sodium (50 mg/kg) by intraperitoneal injection (i.p.), and the spinal cord was exposed at the T9-T10 levels by laminectomy. The SCI was induced by a pneumatic impact device. The force was set at 0.5 m/s, and the duration time was 80 ms. After surgery, bladders were manually voided twice a day until the mice could urinate normally. The mice were randomly divided into sham, sham+TUDCA, SCI, and SCI+TUDCA groups. The mice in sham and sham+TUDCA groups were only subjected to surgical procedures without SCI. Refer to previous reports, TUDCA at 200 mg/kg dosage was given to the mice by oral route once daily post-injury [24, 25]. Meanwhile, an equal volume of saline was given orally once daily in sham and SCI groups.

### Functional behavior evaluation

Hindlimb motor function was evaluated using Basso-Beattie-Bresnahan (BBB) locomotion scale and footprint test performed at different time points. The BBB scale ranges from 0 to 21 (0 = complete paralysis to 21 = normal gait) based on hindlimb joints movement and coordination. The footprint test was performed by dipping the posterior limb of the mice with black dye. Then, the mice were encouraged to walk straight to across a narrow path to record the footprints. The footprints were scanned, and the digitized images were used to analyze their gaits.

### Tissue preparation

The mice were sacrificed by cervical dislocation under anesthetization at specific time points accordingly. For western blot, the spinal cord tissues around the lesion epicenter (± 0.5cm) were isolated and then homogenized in RIPA buffer (Beyotime, Jiangsu, China) with 10 μl/ml protease inhibitor cocktail. The tissue lysates were centrifuged at 12,000 rpm for 15 min at 4°C, and then, the supernatants were collected. A protein assay kit (BCA, Beyotime, Jiangsu, China) was used to determine the protein concentration.

For immunofluorescent staining and histological assessment, mice were transcardially perfused with 4% paraformaldehyde (PFA in 0.1 M PBS, pH 7.4) under anesthetic after normal saline perfusion. Spinal cord tissues were dissected out and fixed with 4% paraformaldehyde overnight. Tissues were dehydrated sequentially with 70%, 80%, 95%, and 100% ethanol. Then, the processed tissues were embedded in paraffin with an appropriate orientation. The paraffin-embedded spinal cord was sectioned at 5 μm using a microtome, and sections were mounted onto histological glass slides and dried overnight for immunostaining or stored at room temperature until use.

### Western Blotting analysis

Equal amounts of total protein from tissue homogenates were separated on SDS-PAGE gels and transferred onto PVDF membranes (Millipore, USA). After blocking with 5% skimmed milk in 0.1% Tween 20 in Tris buffer solution (TBST) for 1 h at room temperature, the membranes were incubated with primary antibodies against Nrf2 (1:1000, R&D Systems), NADPH quinine oxidoreductase-1 (NQO-1, 1:1000, Abcam), glial fibrillary acidic protein (GFAP, 1:1000, Abcam), growth-associated protein 43 (GAP43,1:1000, NOVUS), myelin basic protein (MBP, 1:1000, NOVUS), and β-tubulin (1:1000, CST) overnight at 4°C. And then the membranes were incubated with horseradish peroxidase-conjugated IgG antibody at room temperature for 1 h. Immunoreactive bands were visualized by a ChemiDoc^TM^ MP Imaging System (Bio-Rad), and the integrated density for each band was quantified with Image J software (National Institutes of Health, Bethesda, MD).

### Immunofluorescent staining on cells and sections

The primary cortical neurons cultured on the coverslips were fixed at defined time points with 4% PFA for 2 h at 4°C. The sections were dewaxed in xylene three times each for 5 min and re-hydrated through a series of alcohol with descending concentrations (100 to 95% to 80 to 70% and then tap water twice, each step for 5 min). The prepared tissue sections and cells were blocked in PBS with 10% normal horse serum at room temperature for 1 h and then incubated at 4°C overnight with the following primary antibodies in 10% normal horse serum: microtubule-associated protein 2 (MAP2, 1:200, Boster Biological Engineering Co.), Tuj1 (1:200, Millipore), GFAP (1:500, Boster Biological Engineering Co.), GAP43 (1:200, NOVUS), MBP (1:200, NOVUS), ionized calcium binding adaptor molecule 1 (Iba-1, 1:200, CST), CD163 (1:30, Santa Cruz), and CD68 (1:300, Boster Biological Engineering Co.). The secondary antibodies conjugated with Alexa fluor® fluorochrome (1: 300) were used to detected corresponding primary antibodies. The immunostaining results were checked under a fluorescence microscope (Olympus IX73).

### TUNEL assay

Apoptotic cells in tissue sections were identified by TUNEL staining with cell apoptosis detection kit (Yeasen Biotech, Shanghai, China) following the manufacturer’s protocol. The immunofluorescent images were captured under a fluorescence microscope (Olympus IX73). After TUNEL labeling, the numbers of apoptotic cells (TUNEL positive cells) and the total number of cells (DAPI positive cells) on each section were counted.

### Statistical analysis

All statistic results are presented as the means ± SD. Statistical analysis was performed using one-way ANOVA for the comparison with more than two groups, or using unpaired Student’s *t*-test to compare two groups, with GraphPad Prism 8 software (GraphPad Software Inc.). For statistical analysis, *p*<0.05 was considered statistically significant (expressed as *p<0.05 or **p<0.01).

### Sholl analysis

Sholl analysis was performed to analyze the number of branch intersection with Image J software as previously described [26]. Statistics were calculated using one-way ANOVA with Bonferroni’s multiple comparisons test.

## Results

### TUDCA attenuated H_2_O_2_-induced axon degeneration in cortical neurons

**Fig. 1:**
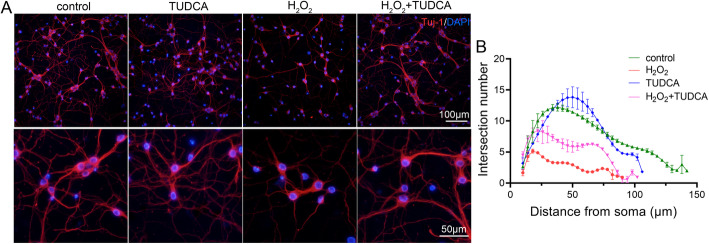
TUDCA alleviated axon degeneration caused by H_2_O_2_ treatment. **A** Immunofluorescence images showing the axon labeled with Tuj1 in primary cortical neurons. **B** Sholl analysis of the axon from the cell body. Statistics were calculated using one-way ANOVA with Bonferroni’s multiple comparisons test. Data were presented as mean ± SD, *n*=3

TUDCA has been reported to protect various cell types from oxidative stress [27]. We found that TUDCA protected mouse cortical neurons from oxidative stress by reducing ROS generation and LDH release caused by H_2_O_2_ treatment and restoring SOD activity (Figure S1). We also found that TUDCA was nontoxic to mouse cortical neurons. Refer to previous reports [28, 29], TUDCA at 200μM was used in further experiments. Oxidative stress has been demonstrated to contribute to axon degeneration in numerous neurological disorders [30]. As our results showed that TUDCA could reduce oxidative stress, thus we want to ascertain whether TUDCA could attenuate axon degeneration induced by oxidative stress. After treated with 300μM H_2_O_2_ or H_2_O_2_ plus 200μM TUDCA for 24h, immunofluorescent staining of Tuj1 (a marker of neurons) was implemented to evaluate the effect of TUDCA on axon degeneration in primary cortical neurons (Fig. 1A). Sholl analysis was performed to quantify the number of branch intersection and the length of axon. As shown in Fig. 1B, TUDCA significantly increased the number of branch intersection and the length of the axon, which suggested that TUDCA treatment attenuated H_2_O_2_-induced axon degeneration in primary cortical neurons as compared with control.

### TUDCA reduced tissue damage and improved motor function after SCI

It has been reported that TUDCA can penetrate through the blood-brain barrier without any neuro- and cyto-toxicity. To determine the effect of TUDCA in normal mice, footprint test (Figure S2A), H&E staining (Figure S2B), Nissl staining (Figure S2C), and immunostaning of GFAP and MAP2 (Figure S3) were performed to evaluate the motor function, histological morphology, and the distribution of astrocytes and neurons. There was no obvious difference between sham and sham+TUDCA groups.

**Fig. 2:**
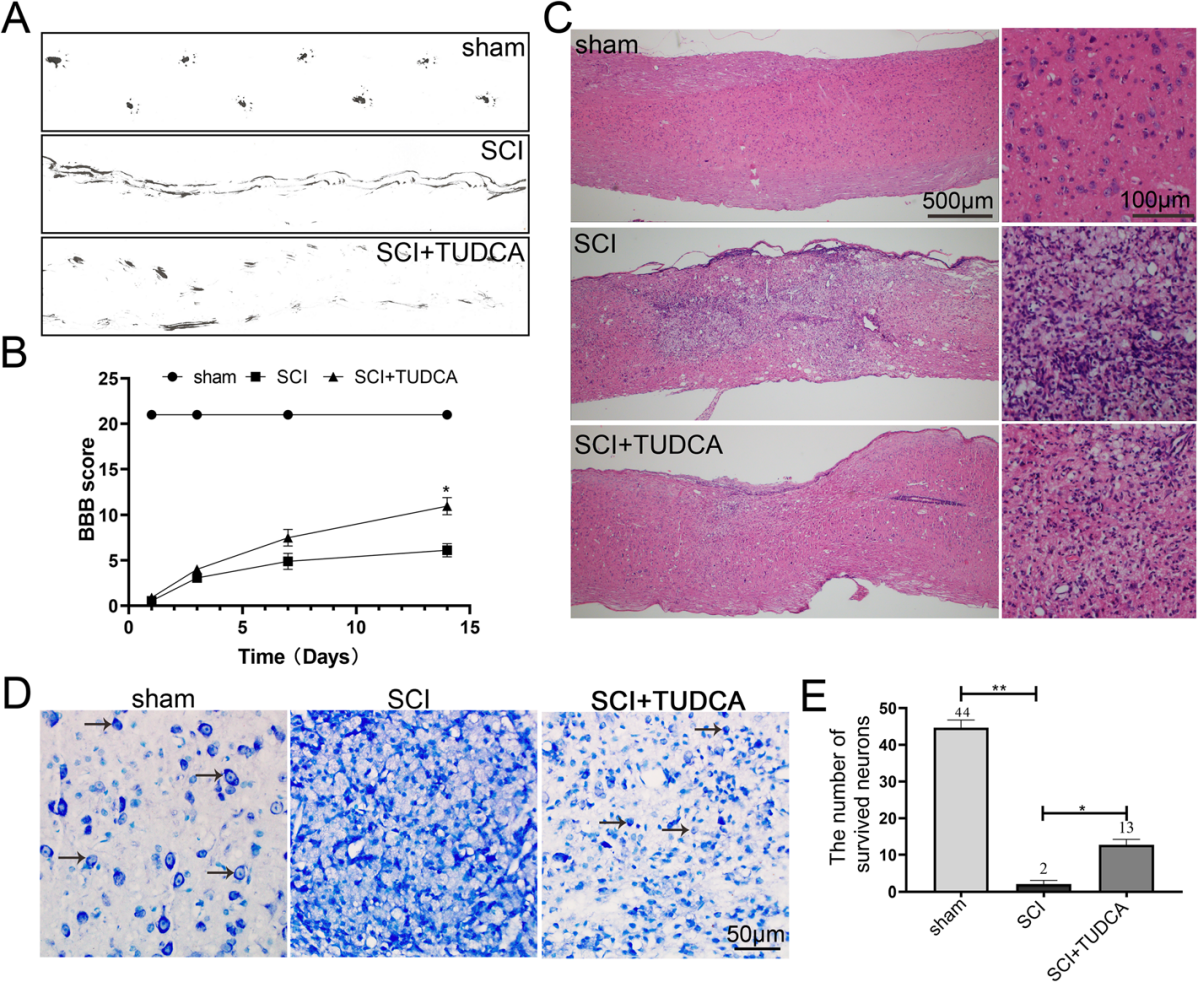
TUDCA improved pathology and motor function after SCI. **A** Footprint analyses of the different groups at day 14 post-injury. **B** The Basso-Beattie-Bresnahan (BBB) locomotion scores of the different groups. **C** Representative images from H&E staining in longitudinal section 14 days after injury. **D**, **E** The survived neurons were stained by Nissl Staining from different groups. All experiments were performed in triplicated and data were presented as means ± SD, *n*=3 per group. *P < 0.05, **P < 0.01

To determine the effect of TUDCA treatment in functional recovery of SCI mice, we performed footprint test (Fig. 2A) and Basso-Beattie-Bresnahan (BBB) rating scale (Fig. 2B). The footprint test revealed that mice in the SCI group showed inconsistent behavior with extensive dragging, while SCI mice treated with TUDCA showed a relatively consistent posterior limb footprint at day 14 after SCI (Fig. 2A). The BBB scores decreased in SCI and SCI+TUDCA groups at 1, 3, 7, and 14 days comparing with the sham; however, at day 14, SCI mice treated with TUDCA showed higher BBB scores than those in the SCI group (Fig. 2B). H&E staining was performed to elaborate the histological morphology of the injured spinal cord 14 days after SCI. Compared with the sham group, obvious malformation and cavity were observed in the injured site of the spinal cord with SCI (Fig. 2C). After being treated with TUDCA for 14 days, the lesion area significantly decreased and less damaged tissue was observed. Moreover, normal neurons were found in the sham group by Nissl staining. But in the SCI group, there were only a few neurons with normal Nissl at the injured site of the spinal cord. However, the number of neurons in the lesion area was significantly increased after TUDCA treatment (Fig. 2D, E). Consistent with our assumption, TUDCA treatment could reduce tissue damage, protect neurons in the lesion area, and improve the functional recovery of SCI mice.

### TUDCA attenuated oxidative stress through Nrf2 signaling pathway after SCI

**Fig. 3:**
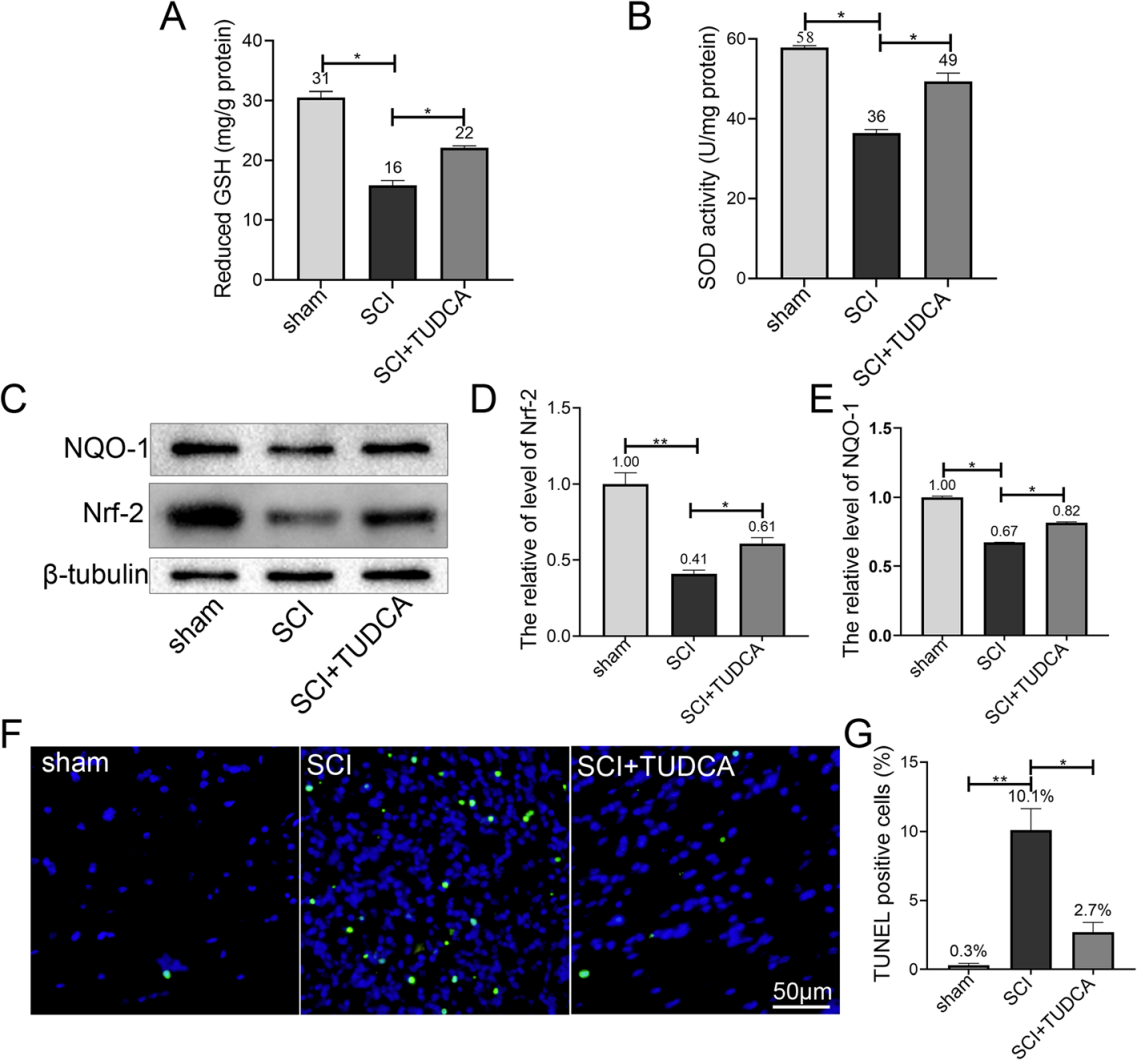
TUDCA exerted neuroprotective effects after SCI through Nrf2/NQO-1 signaling pathway. **A**, **B** The levels of reduced GSH and SOD activity were measured at day 7 post-SCI. **C**–**E** Western blot analysis and quantification of Nrf2, NQO-1 expression at day 7 after SCI. **F**, **G** TUNEL staining was performed to analyze apoptosis 14 days after SCI. All experiments were performed in triplicated and data were presented as means ± SD, *n*=3 per group. *P < 0.05, **P < 0.01

Oxidative stress has been reported to participate in secondary injury progressing after SCI. We determined the level of reduced GSH and SOD activity to evaluate the effect of TUDCA on oxidative stress in SCI mice in vivo. The level of reduced GSH and SOD activity decreased significantly 3 days after SCI, while TUDCA treatment restored the level of reduced GSH and SOD activity (Fig. 3A, B). Nrf2, the “master regulator” of cellular resistance to oxidants was detected by western blot. The result showed that expression of Nrf2 and NQO-1 were increased in SCI+TUDCA group as compared with those in SCI group on day 3 (Fig. 3C–E). These results indicated that TUDCA treatment increased Nrf2 and NQO-1 expression, and suggested that the antioxidant response was activated. In addition, TUNEL staining was performed to evaluate the apoptosis after SCI. As shown in Fig. 3F, only a few TUNEL-positive cells were observed at the spinal cord in the sham group, while the number of apoptotic cells at the lesion sites of the spinal cord increased significantly 14 days after SCI. However, TUDCA treatment could remarkably reduce the cell apoptosis after SCI (Fig. 3G).

### TUDCA decreased the damage of tissue and promoted axon regeneration after SCI

**Fig. 4:**
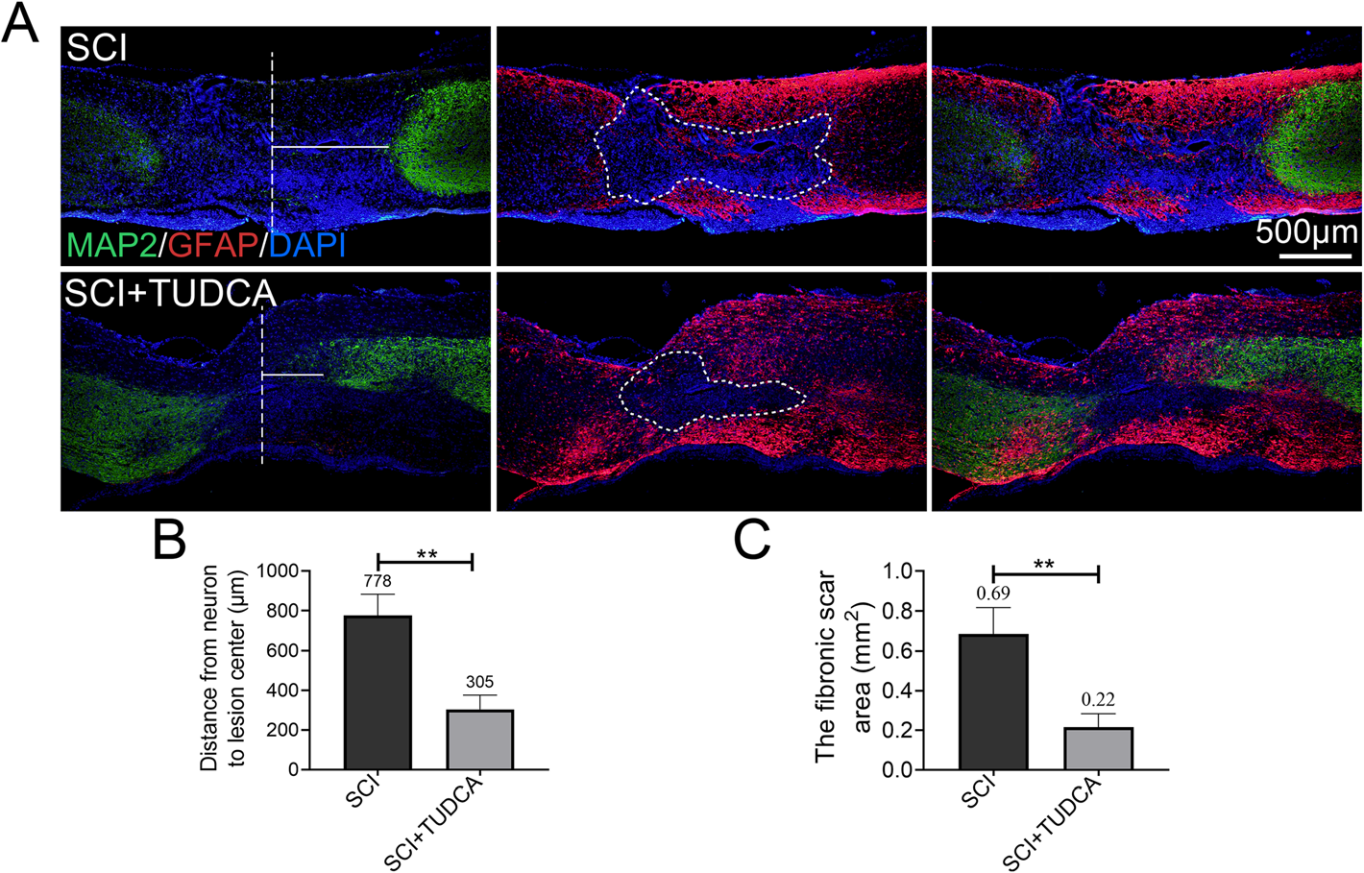
TUDCA decreased the damage of tissue and neurons after SCI. **A** Co-immunofluorescence images showed GFAP (red) and MAP2 (green) 14 days after SCI. **B** Quantification of the distance from neurons to the lesion center from MAP2 immunofluorescence. **C** Quantification of the fibrotic scar surrounding by reactive astrocytes of spinal cord from GFAP immunofluorescence. **P < 0.01

Glial scar and axonal generation in the injured spinal cord is critical to restore motor function after SCI, so we performed immunofluorescent staining and western blot to elaborate the glial scar and axonal regeneration in SCI mice after TUDCA treatment. MAP2 is a neuron-specific cytoskeletal protein that is enriched in dendrites and is used to label the mature neurons. Spinal cord injury leads to the mature neuron loss in or around the lesion site. Immunostaining of MAP2 was used to measure the distance from the lesion center to the nearest neuron. Compared to the SCI group, the distance of injured spinal cord treated with TUDCA significantly decreased (Fig. 4A, B). Following the SCI, astrocytes were activated and migrated to the lesion site to create a barrier surrounding the lesion site around 14 days post-SCI. As shown in Fig. 4A, an obvious glial scar surrounded by activated astrocytes was observed in the SCI groups, and MAP2-positive axons were not observed in the lesion site surrounded by GFAP-positive astrocytes. The glial scar area was measured by Image J (Fig. 4C). Compared with the SCI group, the glial scar area significantly decreased in the SCI+TUDCA group, and the border of the glial scar surrounded by activated astrocytes was not as obvious as the SCI group (Fig. 4A). Moreover, western blot assay showed the GFAP expression was downregulated in SCI+TUDCA groups. This indicated that TUDCA treatment inhibited reactive astrogliosis to prevent astrocytes from forming an overlapping wall of densely packed and adhered cells in the lesion penumbra.

**Fig. 5:**
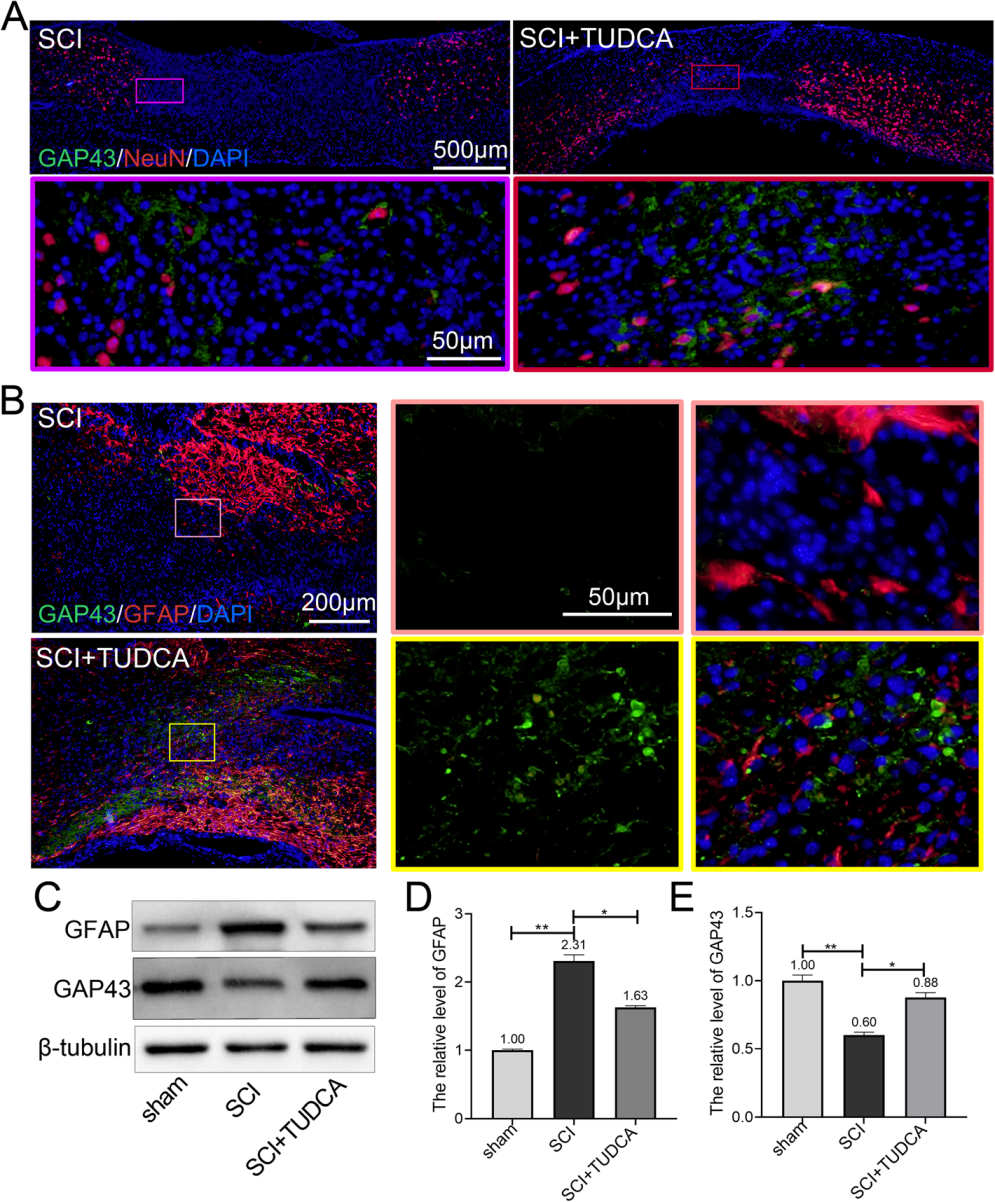
TUDCA promoted axonal regeneration after SCI. **A** Co-immunofluorescence images showed the loss of neurons NeuN (red) and axon regeneration GAP43 (green) in the lesion site 14 days after SCI. **B** Co-immunofluorescence images showed the axonal regeneration (GAP43, green; GFAP, red) in the fibrotic scar on day 14 post-SCI. **C**–**E** Western blot analysis and quantification of GFAP and GAP43 expression. All experiments were performed in triplicated and data were presented as means ± SD, *n*=3 per group. *P < 0.05, **P < 0.01

Double immunofluorescent staining of NeuN and GAP43 (Fig. 5A), GFAP and GAP43 (Fig. 5B) were performed respectively to observe the axon regeneration in the lesion site. GAP43 is closely related to nerve regeneration and plays a key role in guiding axon growth and regulating axon formation of new connections. Loss of neurons at lesion site was observed in the SCI and SCI+TUDCA groups, while reduced the neuron loss region was found in the SCI+TUDCA group comparing with the SCI group (Fig. 5A). The regenerated GAP43-positive axons were found along and in front of neurons. In the SCI group, a few GAP43-positive axons were observed in the fibrotic scar surrounded by activated astrocytes, whereas more GAP43-positive axons were observed in the SCI+TUDCA group as compared with the SCI group (Fig. 5B). The western blot results revealed that GAP43 expression was restored after TUDCA treatment (Fig. 5C, E). In summary, these results indicated that TUDCA treatment elicited the axon outgrowth.

### TUDCA treatment was able to promote remyelination in the lesion site of SCI.

**Fig. 6:**
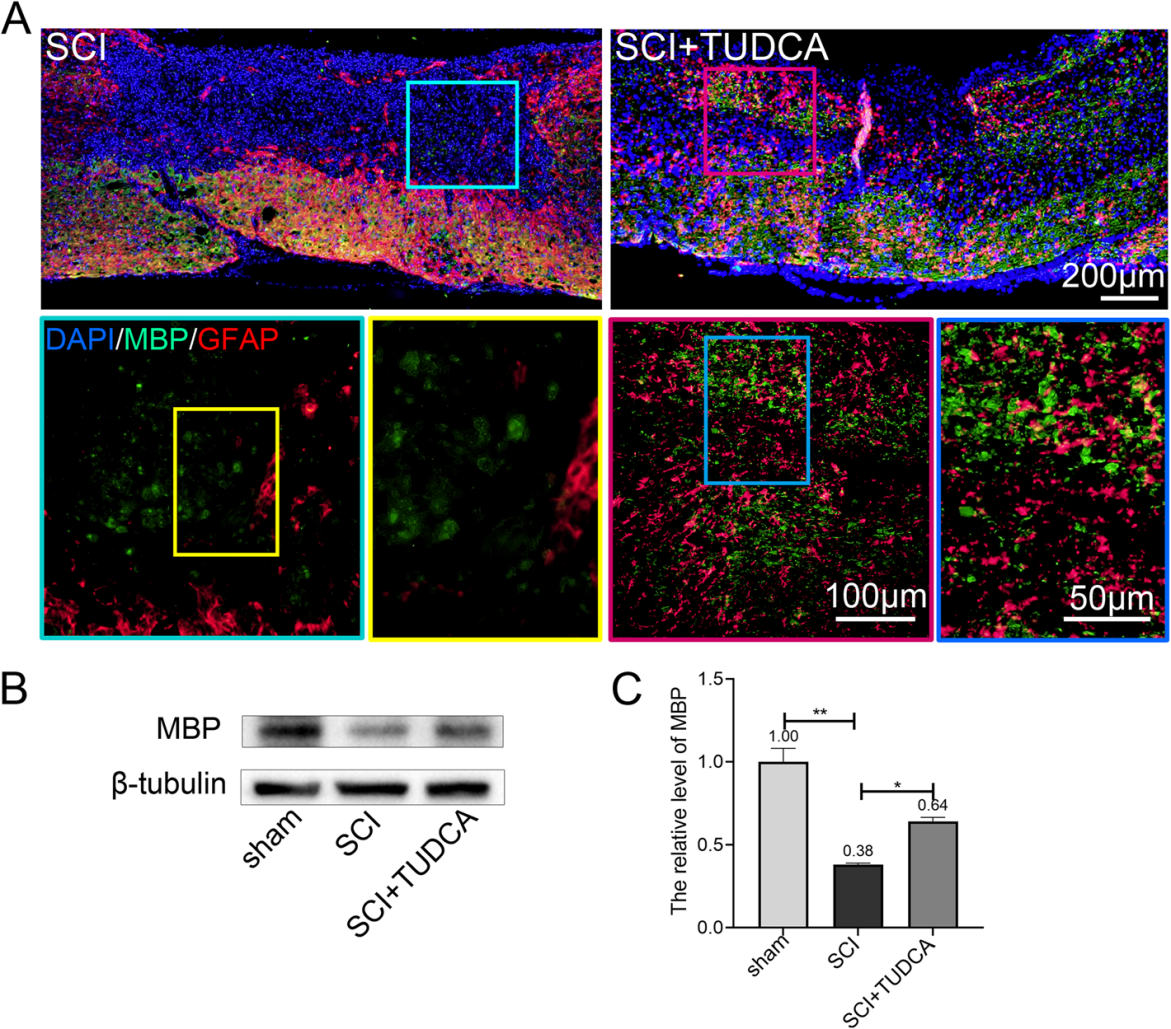
TUDCA treatment promoted remyelination. **A**. Immunofluorescent images of spinal cord on day 14 post-SCI showing the distribution of MBP (green) and GFAP in the lesion site. **B**, **C** Western blot analysis and quantification data of MBP expression in each group. All experiments were performed in triplicated and data were presented as means ± SD, *n*=3 per group. *P < 0.05, **P < 0.01

After SCI, the secondary damages caused necrosis and apoptosis of oligodendrocytes which lead to axonal demyelination [31]. Myelin plays a key role in maintaining the integrity of axons [32]. Therefore, remyelination is important for the functional recovery of axons after SCI. Myelin basic protein (MBP) is an indispensable and structural hydrophilic protein of myelinated axons, which has been used to identify myelinated axons and active remyelination. Therefore, MBP and GFAP double immunofluorescent staining was performed to detect the remyelination in the lesion site 14 days after SCI. In the SCI group, lower MBP expression was observed in the fibrotic scar surrounded by activated astrocytes at the lesion site. But in the SCI+TUDCA group, MBP expression increased significantly in the lesion site of the spinal cord (Fig. 6A). The western blot results also showed that MBP protein expression was restored by TUDCA treatment after SCI (Fig. 6B, C).

### TUDCA promoted microglia polarization toward the M2 phenotype to reduce inflammatory reaction

**Fig. 7:**
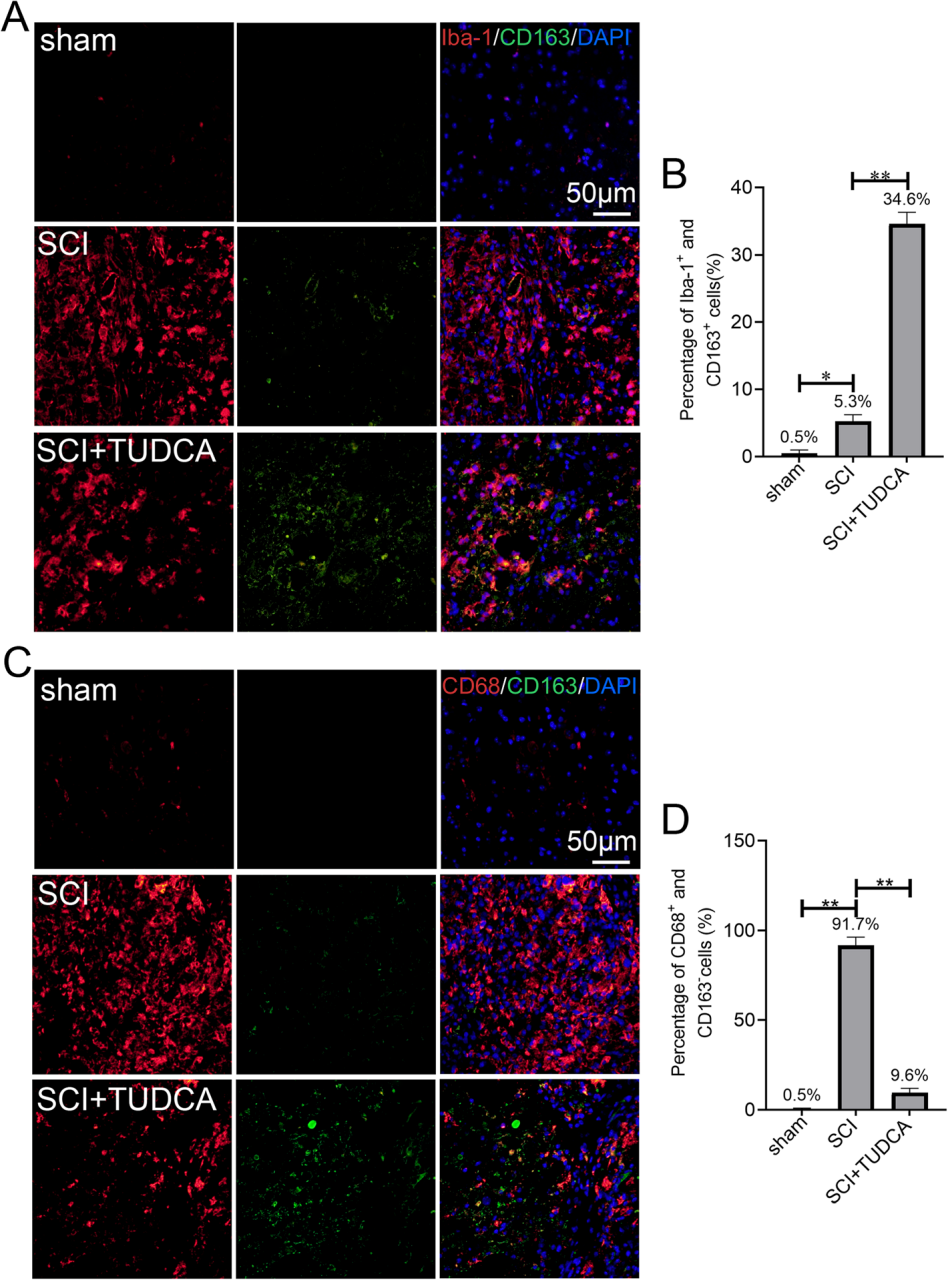
TUDCA treatment promotes microglia polarization toward the M2 phenotype. **A**, **C** Immunofluorescent staining of Iba-1(red) /CD163(green) or CD68(red)/CD163(green) in the lesion site of the spinal cord 14 days after SCI. **B**, **D** Quantification the number of Iba-1^+^/CD163^+^ or CD68^+^/CD163^−^ cells in spinal cord. All experiments were performed in triplicated and data were presented as means ± SD, *n*=3 per group. *P < 0.05, **P < 0.01

Inflammation induces apoptosis around the lesion after SCI [33]. Inhibiting excessive inflammation is essential to prevent secondary damage and promote functional recovery. M1 macrophages have been reported to dominate the lesion site at the early injury and initiate secondary damage. We performed Iba-1 (microglia marker), CD163 (M2-associated marker), CD68 (microglia activation marker), and CD163 double immunofluorescent staining to evaluate the distribution of the activated macrophages/microglia. As shown in Fig. 7, microglia labeled with Iba-1 and CD68 were increased significantly after SCI but decreased with TUDCA treatment, whereas CD163 was increased after TUDCA treatment. Therefore, TUDCA treatment caused macrophages shifting from M1 to M2 phenotype and reduced the number M1 macrophages in the injured spinal cord. These results suggested that TUDCA treatment could regulate the excessive inflammation response which might further promote remyelination and accelerate axonal regeneration.

## Discussion

The pathophysiology of SCI is a complex process that can be generally divided into primary and second injuries. Timely and effective intervention to alleviate secondary injury can effectively reduce further neurological damage and improve functional recovery [5, 34]. In this study, we found that TUDCA could protect primary cortical neurons and improve functional recovery through inhibiting oxidative stress, inflammatory response and apoptosis induced by the primary injury of SCI.

TUDCA, a chemical chaperone, has been reported to possess neuroprotective properties, including anti-apoptosis, anti-inflammation, and anti-oxidative stress in different animal models of neurological diseases [15, 35–37]. It could activate Nrf2 signaling to prevent ROS production and decrease oxidative stress in PD models [16]. TUDCA abolishes unfolded protein response (UPR) activation to ameliorate axonal degeneration in X-linked adrenoleukodystrophy [38]. In a neuroinflammation mouse model, TUDCA showed an anti-inflammation effect through reducing glial cell activation and increasing intracellular cAMP levels in microglia [37, 39]. As a neuroprotective agent with multiple functions, the role of TUDCA in SCI is still unclearly. Therefore, we aim to demonstrate the protective effect of TUDCA on functional recovery and its underlying mechanism using the SCI model.

Oxidative stress has been reported to induce and accelerate secondary injury process in SCI after the physical trauma to the spinal cord [40, 41]. Therefore, suppression of oxidative stress has been considered as an effective therapeutic strategy to improve SCI recovery. First, we checked the protective effect of TUDCA on H_2_O_2_-induced oxidative stress in primary cortical neurons. The results showed that TUDCA increased cell viability by inhibiting ROS production, LDH release, and restoring SOD activity in vitro. Besides, the immunofluorescent staining showed that TUDCA treatment alleviated the axon degeneration which was caused by oxidative stress.

Then, we further investigated whether TUDCA could improve functional recovery in SCI mice. The behavioral test revealed that TUDCA treatment promoted motor functional recovery after SCI. TUDCA treatment significantly reduced the lesion area and increased the number of neurons with normal morphology and Nissl bodies. The level of reduced GSH and SOD activity after SCI was increased by TUDCA, implying that the extent of oxidative stress was limited [41]. Nrf2 is a transcription factor that regulates antioxidating enzyme expression including NQO-1, and glutathione S-transferases (GST) [42]. TUDCA treatment increased Nrf2 and NQO-1 expression after SCI, suggesting that TUDCA inhibited oxidative stress via activating Nrf2 and antioxidant enzymes NQO-1. TUNEL staining showed that TUDCA treatment reduced apoptosis in the lesion site in SCI. Therefore, TUDCA exerted neuroprotective effects by reducing oxidative stress and cell apoptosis after SCI.

At the site of injury, extensive neuronal cell death, axon degeneration and demyelination, and initiation of immune responses would result in more severe secondary injury leading to neural dysfunction after SCI [43]. The glial scar formation helps to control inflammation to a limited extent, but a prominent and permanent glial scar generates a dense barrier which inhibits axon regeneration at the lesion site [44]. Thus, finding an intervention which can block proinflammatory factors and reduce the glial scar area to benefit axon regeneration and remyelination is critical for the functional recovery in treating SCI. Here, we demonstrated the effects of TUDCA on inflammation and glial scar formation in SCI mice. GFAP and GAP43 double labeling was performed to detect the fibrotic component area of the glial scar and the axon regeneration at the lesion site of SCI. Our data showed that at day14 post-SCI, GFAP expression increased significantly, and a glial scar with GFAP-positive astrocytes in the penumbra was formed in the injured spinal cord. We also observed that activated astrogliosis at the inner margin of the lesion penumbra created palisading-like patterns with thick hypertrophied processes that densely overlapped and packed around the lesion in SCI mice, which is consistent with the previous reports [2, 45], while the GFAP expression was remarkably decreased with TUDCA treatment, and the border formed by activated astrocytes in the lesion penumbra was not obvious as in the SCI group. In addition, the fibrotic component area of the glial scar was also reduced. These results revealed that TUDCA treatment reduced the glial scar formation, which could subsequently provide an appropriate micro-environment for axon regeneration. Double immunofluorescent staining of NeuN/GAP43 and western blot showed that TUDCA treatment accelerated the axon regeneration at lesion site.

After SCI, activated microglia/macrophages not only release proinflammatory cytokines that cause cytotoxicity and demyelination but also produce neuroprotective molecules that preserve myelination and stimulate axon regeneration and sprouting [46]. According to the role of microglia/macrophages in SCI, they are divided into two subpopulations, M1 cells release proinflammatory cytokines and M2 cells promote remyelination [47, 48]. Therefore, phase change between M1 and M2 polarizations will lead to a shift between inflammation and remyelination. This is important for functional recovery after SCI [49]. M1 macrophages have been reported to be the dominant macrophage type found at the lesion site [50]. In this study, we found that CD68-positive and CD163-negative M1 macrophages mainly distributed in the fibrotic component of the glial scar, while there were a few cells that were MBP positive. TUDCA treatment significantly reduced the number and distribution of M1 macrophage, along with active remyelination at the lesion site, indicating that TUDCA treatment can mitigate the inflammatory response and enhance the remyelination after SCI.

However, we acknowledged that more detailed mechanisms underlying this TUDCA-mediated neural protection and regeneration are still unclear; hence, further investigation would focus on exploring how TUDCA affects the endogenous neural progenitors and related growth factor secretion during the neural regeneration and remyelination in SCI treatment.

## Conclusion

In conclusion, this study provided evidence showing that TUDCA treatment significantly attenuates secondary injury and improves motor function recovery in SCI mice. TUDCA alleviates oxidative stress through Nrf2/NQO-1 signaling pathway and shifted M1 to M2 macrophages to protect motor neurons and promote axon regeneration and remyelination. Our study sheds light on the beneficial effect of TUDCA on alleviating the second injury of SCI and opens a door of identifying TUDCA-related therapeutic strategy for SCI treatment.

## Supplementary Information

**Additional file 1: Figure S1, Figure S2A–C, Figure S3.**

## Data Availability

All data used in the current study are available from the corresponding author on reasonable request.

## Abbreviations

AD: Alzheimer’s disease
ATP: Adenosine triphosphate
BBB: Basso-Beattie-Bresnahan
CCK-8: Cell Counting Kit-8
FDA: Food and Drug Administration
GAP43: Growth-associated protein 43
GFAP: Glial fibrillary acidic protein
GSH: Glutathione
HD: Huntington’s disease
LDH: Lactate dehydrogenase
LPS: Lipopolysaccharide
MAP 2: Microtubule-associated protein 2
MBP: Myelin basic protein
MPTP: 1-Methyl-4-phenyl-1,2,3,6-tetrahydropyridine
NeuN: Neuronal nuclei
NQO-1: NADPH quinine oxidoreductase-1
Nrf2: Nuclear factor erythroid 2-related factor 2
PD: Parkinson’s disease
PFA: Paraformaldehyde
ROS: Reactive oxygen species
SCI: Spinal cord injury
SOD: Superoxide dismutase
TUDCA: Tauroursodeoxycholic acid
Tuj1: Neuron-specific class III beta-tubulin
UPR: Unfolded protein response
