# Supplementary material for: Tauroursodeoxycholic acid alleviates secondary injury in spinal cord injury mice by reducing oxidative stress, apoptosis, and inflammatory response
Source: J Neuroinflammation. 2021 Sep 20;18:216. doi: 10.1186/s12974-021-02248-2 (PMC8454169; doi:10.1186/s12974-021-02248-2)
Supplement: Supplementary file 1 — Additional file 1: Figure S1, Figure S2A–C, Figure S3. [file 12974_2021_2248_MOESM1_ESM.docx]

**Supplementary figures**


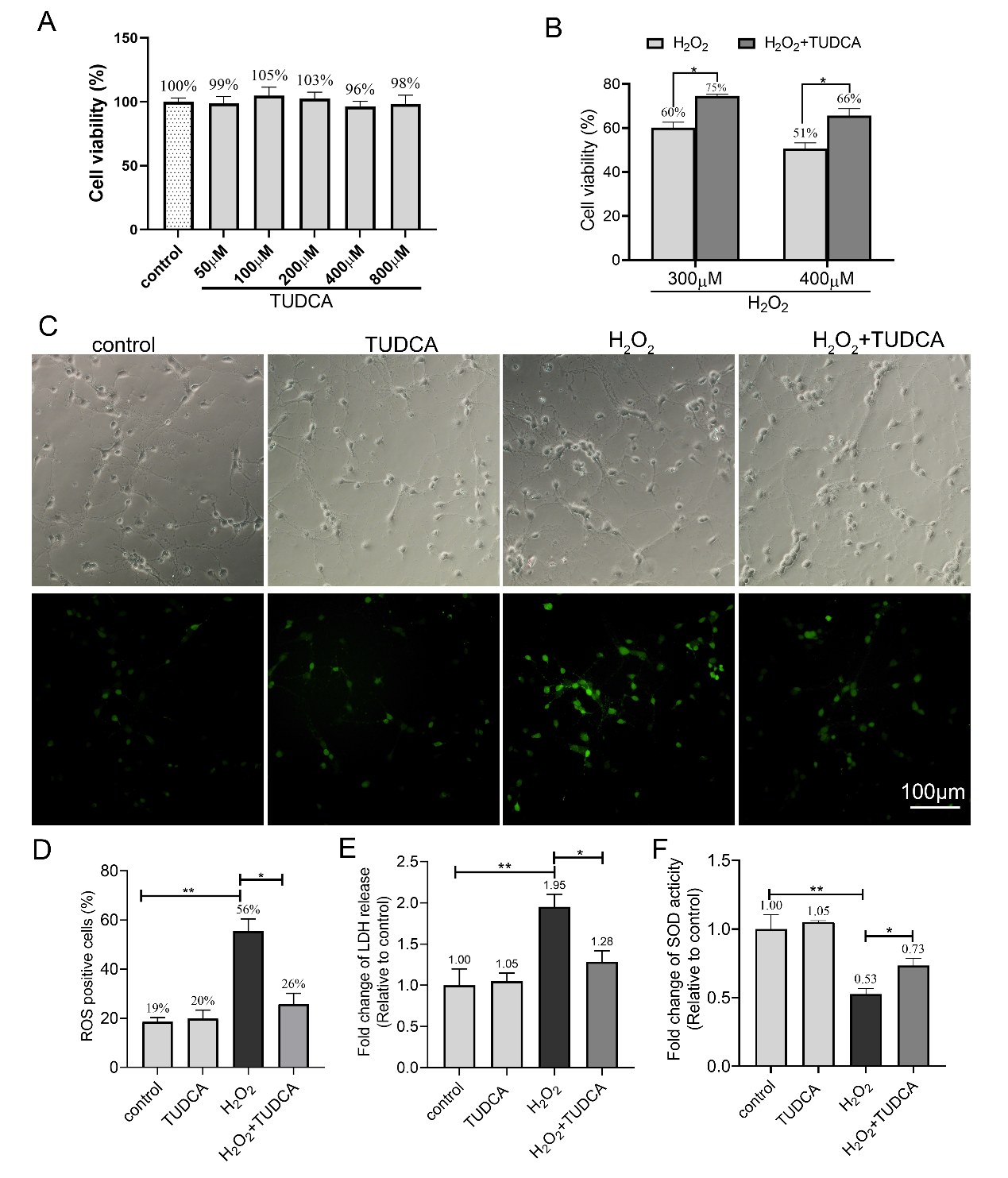


**Figure S1. TUDCA protected cortical neurons from oxidative stress induced by H_2_O_2_.** (A) Cells were treated with different concentrations of TUDCA for 48h. Cells treated with TUDCA showed similar cell viabilities as control, indicating TUDCA is nontoxic to mouse cortical neurons. (B) Cortical neurons were exposed to H_2_O_2_ and treated with 200μM TUDCA for 24h. Cell viability was measured by CCK-8 assay. H_2_O_2_ treatment caused a significant decrease in cell viability as compared with that in control, while co-treatment with TUDCA significantly increased the cell viability comparing to the cells treated with H_2_O_2_ alone. (C, D) Cortical neurons were treated with 300μM H_2_O_2_ or H_2_O_2_ plus 200μM TUDCA for 24h, intracellular ROS generation was detected using DCFH-DA. TUDCA treatment significantly reduced ROS generation caused by H_2_O_2_. (E, F) Detection of LDH release and SOD activity. TUDCA treatment significantly reduced LDH release caused by H_2_O_2_ treatment and restored SOD activity. All experiments were performed in triplicated and data were presented as means ± SD, n=3. **P* < 0.05, ***P* < 0.01.


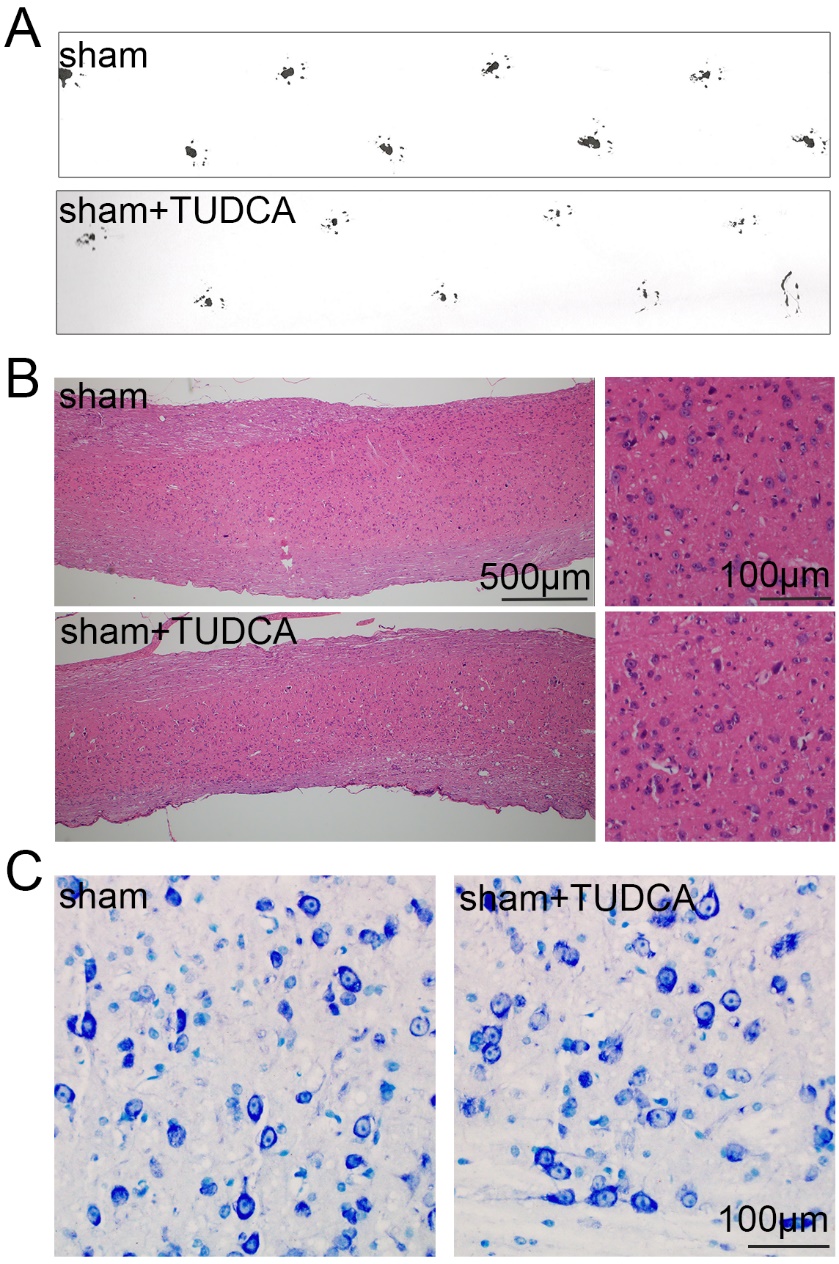


**Figure S2. The motor function, histological morphology, and the normal neurons in normal mice treated with TUDCA.** (A) Footprint analysis of normal mice treated with TUDCA for 14 days. (B) Representative images from H&E staining in longitudinal section. (C) The survived neurons were stained by Nissl Staining.


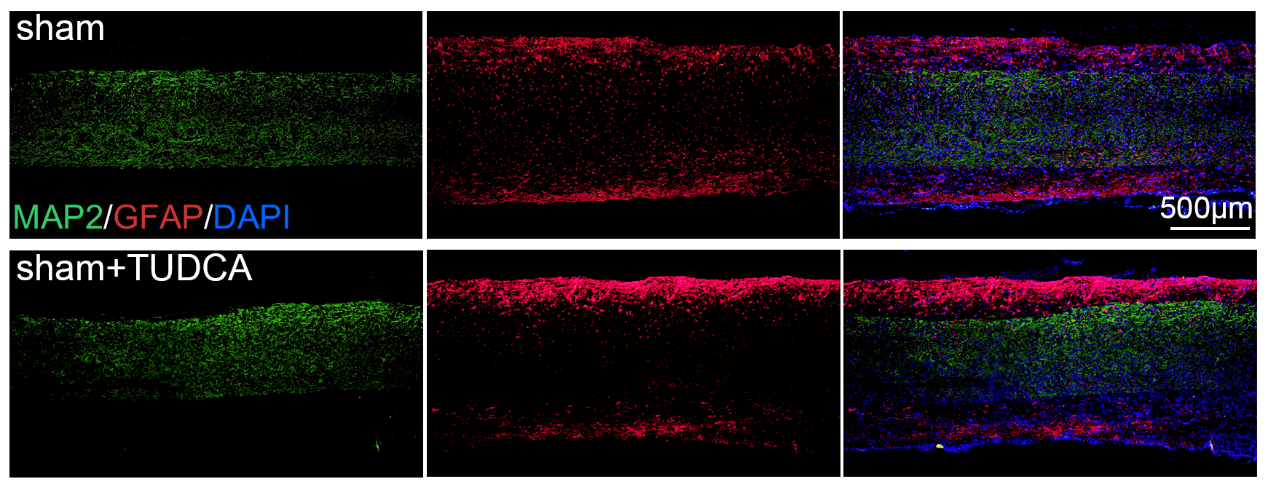


**Figure S3. The distribution of astrocytes and neurons in normal mice treated with TUDCA.** Co-immunofluorescence images showed the distribution of astrocytes (GFAP positive, red) and neurons (MAP2 positive, green) in normal mice treated with TUDCA for 14 days.
